# Bioenergetics and Reactive Nitrogen Species in Bacteria

**DOI:** 10.3390/ijms23137321

**Published:** 2022-06-30

**Authors:** Vitaliy B. Borisov, Elena Forte

**Affiliations:** 1 Belozersky Institute of Physico-Chemical Biology, Lomonosov Moscow State University, Leninskie Gory, 119991 Moscow, Russia; 2 Department of Biochemical Sciences, Sapienza University of Rome, 00185 Rome, Italy; elena.forte@uniroma1.it

**Keywords:** bacterial pathogen, host defense, infectious diseases, human health, molecular bioenergetics, electron transport chain, terminal oxidase, cytochrome oxidase, membrane protein

## Abstract

The production of reactive nitrogen species (RNS) by the innate immune system is part of the host’s defense against invading pathogenic bacteria. In this review, we summarize recent studies on the molecular basis of the effects of nitric oxide and peroxynitrite on microbial respiration and energy conservation. We discuss possible molecular mechanisms underlying RNS resistance in bacteria mediated by unique respiratory oxygen reductases, the mycobacterial *bcc*-*aa*_3_ supercomplex, and *bd*-type cytochromes. A complete picture of the impact of RNS on microbial bioenergetics is not yet available. However, this research area is developing very rapidly, and the knowledge gained should help us develop new methods of treating infectious diseases.

## 1. Introduction

Primary bacterial pathogens are infectious agents responsible for severe and often deadly diseases in humans. In addition, commensal bacteria can produce opportunistic infections in immunosuppressed patients. Disease-causing bacteria are becoming resistant to most commonly available antibiotics, which poses a threat to global public health. The production of reactive nitrogen species (RNS) by the innate immune system is part of the host’s defense against invading microbes. RNS refers to various nitrogenous products including nitric oxide (^•^NO), peroxynitrite anion (ONOO^–^), nitroxyl (HNO), dinitrogen trioxide (N_2_O_3_), nitrite (NO_2_^–^), nitrogen dioxide (^•^NO_2_), nitronium cation (NO_2_^+^), nitrosonium cation (NO^+^), nitrosoperoxycarbonate anion (ONOOCO_2_^–^), nitryl chloride (Cl-NO_2_), S-nitrosothiols (RSNOs) [1]. ^•^NO, along with carbon monoxide and hydrogen sulfide, is considered an endogenous gaseous signaling molecule [2,3,4,5]. ^•^NO is the main RNS produced by the host and the main source for the generation of the other RNS. This small diatomic molecule is a free radical, i.e., with one unpaired electron, and can diffuse easily through biological membranes. The enzymes that produce ^•^NO are NO synthases (NOS). They convert l-arginine and O_2_ into l-citrulline and ^•^NO using NADPH as the electron donor. There are three NOS isoforms: neuronal (nNOS), endothelial (eNOS), and inducible (iNOS). nNOS and eNOS are constitutively expressed whereas iNOS expression is induced by immunological stimuli. The latter occurs predominantly in macrophages and plays an essential role in immune defense. ^•^NO can combine with superoxide radical (O_2_^•−^) produced by the NADPH oxidase at diffusion-controlled rates yielding another RNS, ONOO^−^. Under physiological conditions, ONOO^−^ is in equilibrium with peroxynitrous acid, ONOOH (pK_a_ = 6.8), and local pH affects peroxynitrite reactivity. Both ONOO^−^ and ONOOH are able to cross biological membranes. Peroxynitrite is a potent oxidant and nitrating agent, with a very important role in the destruction of invading pathogens by macrophages, as ONOOH spontaneously homolyzes to hydroxyl radical (^•^OH) and ^•^NO_2_ [6,7]. As they are within bacteria-containing phagolysosomes in macrophages, RNS creates a hostile environment that impairs microbial growth. RNS inhibit DNA replication and bacterial respiration [8]. ^•^NO and ONOO^−^ were reported to damage metalloproteins containing heme cofactors and/or iron-sulfur clusters [9]. Additionally, ^•^NO mediates post-translational modifications through S-nitrosylation of protein thiol groups, and peroxynitrite promotes the nitration of protein tyrosine residues [6,10]. This review focuses on the effects of ^•^NO and ONOO^−^ on bacterial aerobic (O_2_-dependent) respiratory (electron transport) chains, namely on their last component represented by a heme-containing terminal oxidase, in light of recent findings.

We have chosen to focus only on aerobic bacteria because data on the RNS stress response of pathogenic anaerobes remain scarce. For instance, in the case of multidrug-resistant *Klebsiella pneumoniae*, a common cause of hospital-acquired pneumonia, some data on its adaptive response toward oxidative stress are available [11,12] but none addressed the bacterial response to ^•^NO. A search of the genome of *K. pneumoniae* points out the presence of ^•^NO-detoxifying enzymes Hmp and Hcp nonetheless [9]. The intracellular pathogen *Shigella flexneri*, which is the causative agent of bacillary dysentery, was reported to be sensitive to ^•^NO produced *in vitro*; on the contrary, ^•^NO is not required for clearance of the microbes in infected mice or macrophages [13]. A search of the genome of *S. flexneri*, however, indicates putative flavorubredoxin, Hmp and Hcp enzymes are involved in nitrosative detoxification [9]. *Clostridioides difficile* is the cause most implicated in antibiotic-associated diarrhea and severe inflammation of the bowel. This anaerobic enteropathogen encodes a few putative ^•^NO-consuming enzymes, such as two flavodiiron proteins FdpA and FdpF, and Hcp [14,15]. FdpA and FdpF, however, show negligible ^•^NO reductase activity but instead significant O_2_ reductase activity [15,16]. Although there is no data on the specific ^•^NO activity of Hcp, the physiological nitrosating agent S-nitrosoglutathione (GSNO) was reported to induce the expression of *hcp* [17]. This finding indicates that Hcp is involved in ^•^NO resistance.

## 2. Bacterial Aerobic Respiratory Chains

Similar to mitochondria of eukaryotic cells, bacteria contain aerobic respiratory chains. The main function of the chains is to create a proton motive force (PMF), a central energy currency. The well-known mammalian mitochondrial chain is linear [18,19]. It consists of the enzyme complexes I, II, III, and IV (Table 1). The complexes I, III, and IV catalyze the oxidation of NADH by ubiquinone, oxidation of ubiquinol by ferricytochrome *c*, and oxidation of ferrocytochrome *c* by molecular oxygen, respectively. Each redox reaction is coupled to the generation of PMF that can be used further for the production of one more central energy currency, ATP, by ATP synthase (also termed complex V) or for active transport of solutes across the membrane. Complex II (succinate dehydrogenase) belongs to both the respiratory chain and the Krebs cycle. Complex II catalyzes the electron transfer from succinate to ubiquinone but unlike complexes I, III, and IV, the transfer is not coupled to the formation of PMF [20,21]. The bacterial respiratory chains, in contrast to the mitochondrial one, are branched, with different routes of electron transfer depending on the growth conditions [22,23]. As a quinone, bacteria can use not only ubiquinone but also menaquinone, plastoquinone, or caldariellaquinone.

In order to transfer electrons from NADH to quinone, bacteria use three different families of NADH:quinone reductases (dehydrogenases)—NDH-1, NDH-2, and NQR (Table 2). NDH-1 reductases are closely related to the mitochondrial complex I and function as redox-driven proton pumps [24,25]. Both NDH-2 and NQR are unrelated to the canonical complex I. NDH-2 enzymes are non-electrogenic and therefore unable to support PMF [26,27]. NQR reductases operate as redox-driven sodium pumps, i.e., they generate a sodium ion motive force rather than PMF [28,29,30]. The sodium ion motive force, along with PMF and ATP, is the third energy currency used by a few bacteria [31]. Bacteria with more than one NADH:quinone reductase show a preference for one or another enzyme depending on the growth conditions.

**Figure 1:**
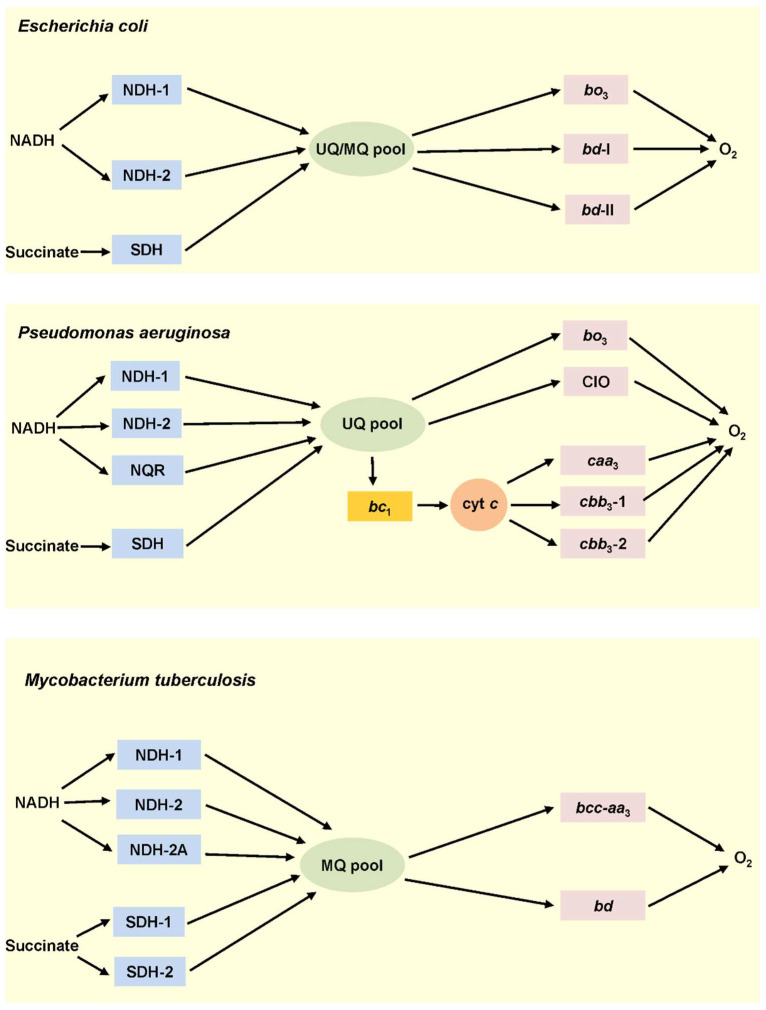
Aerobic respiratory chains of *Escherichia coli*, *Pseudomonas aeruginosa*, and *Mycobacterium tuberculosis*. In *E. coli*, two NADH dehydrogenases, NDH-1 and NDH-2, and succinate dehydrogenase (SDH) transfer electrons to ubiquinone (UQ)/menaquinone (MQ) pool. Three quinol oxidases, cytochromes *bo*_3_, *bd*-I, and *bd*-II, oxidize ubiquinol/menaquinol with the concomitant reduction of O_2_ to 2H_2_O. *P. aeruginosa* has three NADH dehydrogenases, NDH-1, NDH-2, NQR, and SDH. The electrons from ubiquinol are further transferred to O_2_ either directly via two quinol oxidases, cytochrome *bo*_3_ and *bd*-type cyanide insensitive oxidase (CIO), or via the *bc*_1_ complex to three cytochrome *c* oxidases, *caa*_3_, *cbb*_3_-1, and *cbb*_3_-2. *M. tuberculosis* possesses three NADH dehydrogenases, one NDH-1, two NDH-2, and two succinate dehydrogenases, SDH-1 and SDH-2. The electrons from menaquinol are then transferred to O_2_ via cytochrome *bd* or cytochrome *bcc*-*aa*_3_ supercomplex.

Bacterial complex III, also termed cytochrome *bc*_1_ complex, transfers electrons from quinol to ferricytochrome *c*. This redox reaction is coupled with the production of PMF via the Q-cycle (Mitchellian redox-loop) mechanism [32,33]. The presence of complex III in bacterial respiratory chains is optional. Some bacteria, e.g., *Escherichia coli*, have no cytochrome *c* at all, and hence no cytochrome *bc*_1_ [34]. Cytochrome *c* of other bacteria is not water-soluble but fused either to complex III or complex IV. This leads to the formation of a supercomplex between complex III and complex IV (Table 2). Accordingly, the cytochrome *bcc*-*aa*_3_ (III_2_–IV_2_) supercomplex was discovered in *Mycobacterium smegmatis* and *Corynebacterium glutamicum* [35,36,37]. A supercomplex composed of cytochrome *bc*_1_ and *aa*_3_-type cytochrome *c* oxidase was also identified in *Rhodobacter sphaeroides* [38]. Figure 1 shows examples of three different types of branched bacterial respiratory chains in which the complex III is absent (*E. coli* [34]), present as a separate enzyme (*Pseudomonas aeruginosa* [29]), or forms a tight supercomplex with the *aa*_3_-type cytochrome *c* oxidase (*M. tuberculosis* [39,40]).

The membrane-bound terminal oxidases are divided into two superfamilies: heme–copper oxidases and *bd*-type cytochromes [41,42,43]. The active site of a heme–copper oxidase termed the binuclear center (BNC) is composed of a high-spin heme (*a*_3_, *o*_3_, or *b*_3_) and a copper ion (Cu_B_). The enzyme catalyzes the transfer of electrons from cytochrome *c* or quinol to O_2_ with the production of 2H_2_O. The reaction is coupled to the generation of PMF using the mechanism of redox-coupled proton pumping across the membrane [21,22,44,45,46,47,48,49,50,51,52,53,54,55,56,57,58,59]. A heme–copper oxidase that uses cytochrome *c* as an electron donor (cytochrome *c* oxidase) has the second copper site, Cu_A_. Cu_A_ directly accepts electrons from cytochrome *c*. If the enzyme uses quinol as an electron donor (quinol oxidase), Cu_A_ is absent. Heme–copper oxidases also contain a low-spin heme (*a* or *b*) that accepts electrons from Cu_A_ (cytochrome *c* oxidase) or directly from an electron donor (quinol oxidase) and donates them to the BNC. In *caa*_3_ and *cbb*_3_ oxidases, the reduction of Cu_A_ by water-soluble cytochrome *c* is followed by an intermediate reduction of additional heme(s) *c*. The classification of the heme–copper oxidases is based on the organization of the intraprotein proton transfer pathways. Accordingly, the enzymes are divided into three main families: A, B, and C [60,61,62].

The active site of cytochrome *bd* contains a high-spin heme *d* but not a copper ion [39,63,64,65,66,67,68,69]. There are data that one more high-spin heme, *b*_595_, could perform some of the functions of Cu_B_ [70,71,72,73,74,75,76,77,78,79,80,81,82,83,84,85,86]. Similar to heme–copper oxidases, *bd*-type cytochromes couple the reduction of O_2_ to 2H_2_O to the formation of PMF [44,87,88]. However, in contrast to the heme–copper enzymes, cytochromes *bd* do so without being a proton pump [89,90,91]. The lack of proton-pumping machinery decreases the energetic efficiency of the *bd*-type oxidases. Until now, all biochemically characterized cytochromes *bd* turned out to be quinol oxidases [49,92,93,94]. Accordingly, the third heme in cytochrome *bd*, a low-spin *b*_558_, mediates electron transfer from quinol to hemes *b*_595_ and *d*. The *bd*-oxidases typically have a very high affinity for O_2_ and CO due to specific features of heme *d,* which is an iron-chlorin [77,95,96,97,98,99]. In some cases, heme *d* can be replaced with heme *b* [42,100]. Intriguingly, phylogenomic analyses performed by Murali et al. suggest that there are *bd*-type cytochromes that use cytochrome *c* as an electron donor [42]. Phylogenomics by Murali et al. identified three families and several subfamilies within the cytochrome *bd* superfamily. At the same time, earlier classification of the *bd*-type oxidases based on the size of the hydrophilic region between transmembrane helices 6 and 7 in subunit I (a binding domain for quinol oxidation termed the Q-loop) is still commonly used. According to this classification, cytochromes *bd* are divided into two subfamilies: L (long Q-loop) and S (short Q-loop) [101,102].

**Figure 2:**
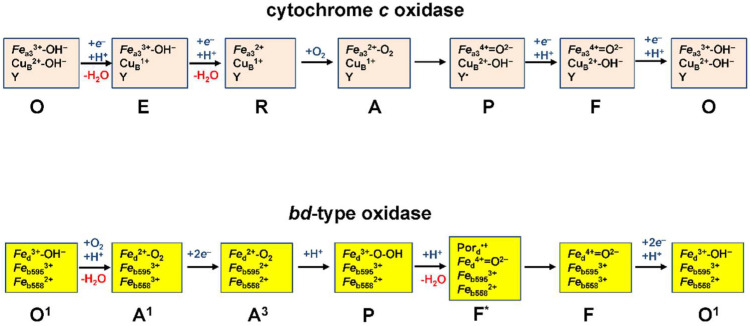
Proposed catalytic cycles of heme–copper cytochrome *c* oxidase and *bd*-type oxidase.

The catalytic cycle of heme–copper oxidases is best studied for the *aa*_3_-type cytochrome *c* oxidases (Figure 2). It includes the intermediates termed O, E, R, A, P, F (see [41] and references therein). The sequential transfer of two electrons to O (the fully oxidized state of the BNC) results in the sequential formation of E and R, one-electron reduced and fully reduced states of the BNC, respectively. R binds O_2_ to produce the A state. Then, the O–O bond is cleaved, and the P state is formed in which heme *a*_3_ is ferryl, Cu_B_ is oxidized, and a conserved tyrosine residue in the BNC is oxidized to a radical, Y^•^. The transfer of the third electron to the BNC re-reduces Y^•^ to Y bringing about the F state. The transfer of the fourth electron to the BNC leads to the reduction of ferryl heme *a*_3_ to ferric form that regenerates the O state and completes the cycle. The O→ E, E→ A, P→ F, and F→ O transitions are electrogenic and coupled to the transfer of a pumped proton (not shown in Figure 2).

The catalytic cycle of *bd*-type oxidases is deduced from the studies on the *E. coli* cytochrome *bd*-I [90,103,104,105,106] (Figure 2). It includes the intermediates termed O^1^, A^1^, A^3^, P, F*, F, and takes into account that the quinol substrate is a two-electron donor. In the O^1^→ A^1^ transition, an electron transfers from heme *b*_558_ to heme *d* and the latter binds O_2_. In the next A^1^→ A^3^ transition, two electrons from a quinol reduce heme *b*_558_ and heme *b*_595_. In the A^3^→ P transition, a true transient peroxy complex of ferric heme *d* is formed concomitant with oxidation of heme *b*_595_. The O–O bond cleavage occurs in the next, P→ F* transition in which the ferric heme *d* is further oxidized to the ferryl form with a porphyrin π-cation radical (Por^•+^). Then in the F*→ F transition, the radical is quenched by an electron from the ferrous heme *b*_558_. The F→ O^1^ transition, in which two electrons from a second quinol reduce the ferryl heme *d* (to the ferric form) and heme *b*_558_, completes the cycle. The P/F*→ F and F→ O^1^ transitions were reported to be electrogenic [88,89,90,91,107].

The key role of most heme–copper oxidases in bacterial metabolism is to create PMF. In the case of cytochromes *bd*, the bioenergetic function is not the only. The *bd* enzymes play other critical roles in microbes [94,108,109,110,111]. They contribute significantly to the ability of bacteria to resist stresses induced by peroxide [49,112,113,114,115,116], sulfide [5,117,118,119,120], ammonia [121], chromate [122], cyanide [117,123]. Due to the fact that the *bd* oxidases are often found in pathogenic bacteria but absent in humans, they can be used as protein targets for next-generation antimicrobials [43,64,68,124,125,126,127,128,129,130,131,132,133,134].

## 3. ^•^NO and Bacterial Terminal Oxidases

### 3.1. ^•^NO and Bacterial Heme–Copper Terminal Oxidases

With the exception of the mycobacterial *aa*_3_-type oxidase (see Section 3.1.1), the bacterial heme–copper oxidases tested to date, such as the *cbb*_3_-type oxidases from *Vibrio cholerae* and *Rhodobacter sphaeroides*, and the *aa*_3_-type oxidase from *R. sphaeroides*, are rapidly and strongly inhibited by ^•^NO [135], similar to their mitochondrial homolog, cytochrome *c* oxidase [136]. The reaction of the mitochondrial enzyme with ^•^NO was studied in more detail. It was shown that low, nanomolar levels of ^•^NO reversibly inhibit the enzyme activity [136] whereas high, micromolar levels of ^•^NO cause irreversible damage to the enzyme [137]. The reversible inhibition occurs via two pathways. At high reductive pressure (high turnover conditions) and low O_2_ tensions, the O_2_-competitive inhibition pathway prevails. It occurs through the reaction of ^•^NO with the two-electron reduced (and possibly one-electron reduced) BNC leading to the production of the nitrosyl derivative of the enzyme. At low reductive pressure (low turnover conditions) and high O_2_ tensions, the noncompetitive pathway prevails. The latter proceeds via reaction of ^•^NO with the catalytic intermediates that have Cu_B_ oxidized, resulting in the generation of the nitrite-bound enzyme [138,139,140,141]. It is reasonable to assume that the bacterial heme–copper oxidases studied [135] are inhibited by ^•^NO through similar mechanisms.

#### 3.1.1. ^•^NO-Metabolizing Activity of the Mycobacterial *bcc-aa*_3_ Supercomplex in Turnover

**Figure 3:**
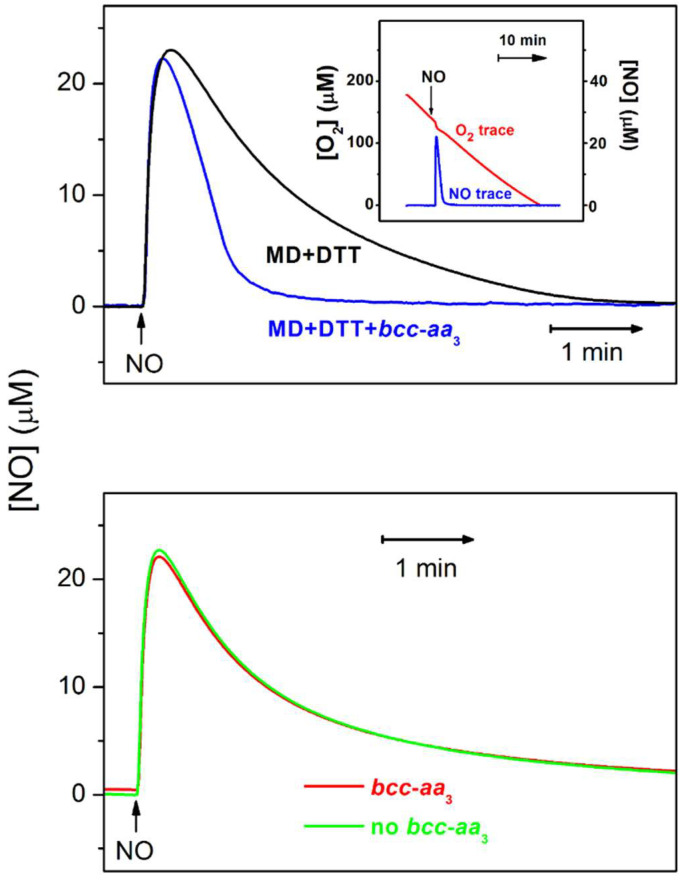
Purified mycobacterial cytochrome *bcc-aa*_3_ supercomplex scavenges ^•^NO under turnover conditions. ***Top panel:*** the *bcc-aa*_3_ in turnover with 5 mM DTT and 0.26 mM MD accelerates the decomposition of 30 µM ^•^NO added. (***Bottom panel)*** in the absence of DTT and MD, i.e., under non-turnover conditions, the *bcc-aa*_3_ does not accelerate the decomposition of 30 µM ^•^NO added. (***Inset***) the effect of 30 µM ^•^NO on the O_2_ consumption by the *bcc-aa*_3_. The Figure was modified from Forte et al. [57] under the terms of the Creative Commons Attribution 4.0 International License.

Mycobacteria contain no water-soluble cytochrome *c*. Probably for this reason their *aa*_3_-type cytochrome oxidase needs to be in a tight supercomplex with cytochrome *bcc*, a homolog of the mitochondrial cytochrome *bc*_1_ [35,36]. Forte et al. reported that a purified chimeric supercomplex composed of *M. tuberculosis* cytochrome *bcc* and *M. smegmatis aa*_3_-type oxidase resists inhibition by ^•^NO [57]. The effect of ^•^NO on the O_2_ consumption by the *bcc*-*aa*_3_ supercomplex in the presence of excess dithiothreitol (DTT) and menadione (MD) was evaluated amperometrically. A very small, short-term decrease in the O_2_ consumption induced by ^•^NO is followed by quick and complete restoration of the initial enzyme’s activity (Figure 3, *inset*). Surprisingly, the ^•^NO decay allowing for the activity recovery occurs much faster than one would expect. The reason for this turned out to be the ability of the *bcc*-*aa*_3_ supercomplex to degrade ^•^NO under turnover conditions. The rate of ^•^NO decay in the presence of the enzyme and reductants is significantly higher than in the presence of the reductants only (Figure 3, *top panel*). Furthermore, in the absence of DTT and MD, the kinetic profiles of ^•^NO decay in aerobic solution with and without the *bcc-aa*_3_ are identical (Figure 3, *bottom panel*). The latter two observations support the conclusion that the ^•^NO decomposition is indeed catalyzed by the purified *bcc*-*aa*_3_ supercomplex in turnover with O_2_ and the electron donors. The maximum ^•^NO-consuming activity of the enzyme measured following the addition of 30 µM ^•^NO appeared to be about 300 mol ^•^NO × (mol *bcc-aa*_3_)^−1^ × min^−1^ [57] (Table 3).

**Figure 4:**
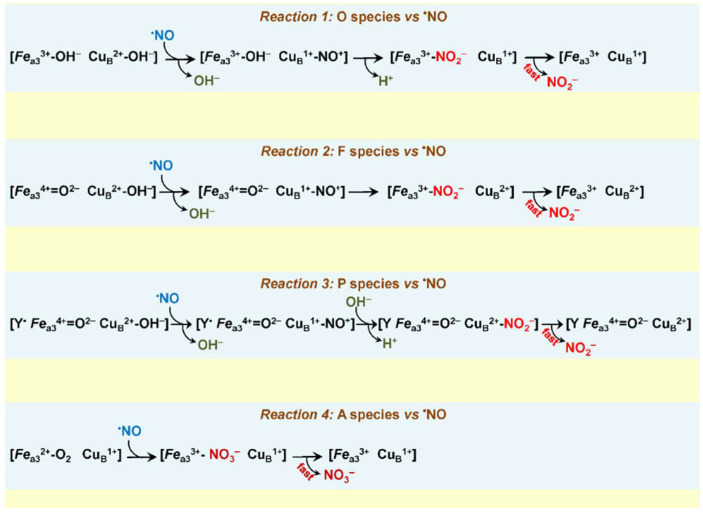
Possible mechanisms of the ^•^NO detoxification catalyzed by the mycobacterial cytochrome *bcc-aa*_3_ supercomplex under turnover conditions. Y in *Reaction 3*—a conserved tyrosine residue in the BNC.

Possible mechanisms for this reaction catalyzed by the *bcc*-*aa*_3_ are worth discussing. Earlier, it was reported that in the mitochondrial cytochrome oxidase, ^•^NO can react with the catalytic intermediates O, P, and F, each according to a 1:1 stoichiometry [138,140]. One could suggest that in the *bcc*-*aa*_3_ ^•^NO also reacts with these species populated at a steady-state. In view of the fact that in the *bcc*-*aa*_3_ the ^•^NO/O_2_ stoichiometry was estimated to be 2.65 [57] i.e., >1, we assume that in this enzyme ^•^NO can react with more than one intermediate during the catalytic cycle. Figure 4 shows possible reaction pathways for the *bcc*-*aa*_3_ taking into account modern views on the structures of intermediates O, F, and P. As in the mitochondrial enzyme [138,140], in the reactions with O, F, and P, ^•^NO is thought to donate one electron to Cu_B_^2+^ yielding nitrosonium ion (NO^+^) and Cu_B_^1+^. This results in the oxidation of ^•^NO to NO_2_^–^ and the conversion of a corresponding intermediate into the succeeding one along the catalytic cycle of the *bcc*-*aa*_3_ (Figure 4, reactions 1, 2, 3, see also Figure 2). In other words, following the reaction with one molecule of ^•^NO, O is converted into E, F—into O, and P—into F. In the mitochondrial cytochrome oxidase, NO_2_^–^ produced from ^•^NO binds with a relatively high affinity to the oxidized heme *a*_3_ (or Cu_B_) in the BNC [140]. This impedes the complete reduction of the BNC and, hence, its ability to bind and further reduce O_2_. As a result, O_2_ consumption is halted. We hypothesize that in the case of the *bcc*-*aa*_3_ NO_2_^–^ generated from ^•^NO does not bind to the BNC with high affinity. Instead, NO_2_^–^ is quickly ejected into the bulk phase from the supercomplex without affecting the catalytic O_2_ consumption.

Since the *bcc*-*aa*_3_ is an O_2_-binding heme protein, it cannot be ruled out that the enzyme is also capable of acting as a ^•^NO dioxygenase. A possible mechanism of such reaction similar to that reported for the truncated hemoglobin N of *M. tuberculosis* [144] is shown in Figure 4 (reaction 4). According to the proposed pathway, the reaction of the catalytic intermediate A with ^•^NO yields nitrate (NO_3_^–^) that should leave the BNC rapidly in order to avoid inhibition of the main O_2_ reductase activity. All proposed reaction mechanisms (Figure 4, reactions 1–4) await experimental confirmation.

#### 3.1.2. ^•^NO Reductase Activity of Heme–Copper Oxidases

**Figure 5:**
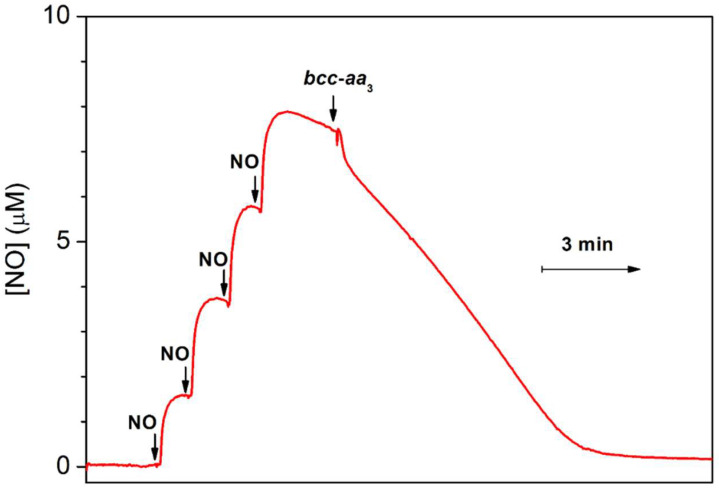
^•^NO reductase activity of the mycobacterial cytochrome *bcc-aa*_3_ supercomplex. Four aliquots of 2.1 µM ^•^NO were sequentially added to degassed buffer containing 5 mM DTT, 0.26 mM MD, 5 mM glucose, and 16 units/mL glucose oxidase. Then, the pre-reduced cytochrome *bcc-aa*_3_ (200 nM) was added. The Figure was modified from Forte et al. [57] under the terms of the Creative Commons Attribution 4.0 International License.

The amperometric studies showed that a few bacterial heme–copper oxidases are able to decompose ^•^NO under reducing anaerobic conditions at ^•^NO concentrations in the solution in the range of 5 to 10 µM. Figure 5 demonstrates such activity of the purified mycobacterial *bcc-aa*_3_ supercomplex [57]. The pre-reduced enzyme was anaerobically added to an O_2_-free solution of ^•^NO in the presence of excess DTT and MD. The addition of the enzyme was shown to increase the rate of the decomposition of ^•^NO. It has to be noted that the slow ^•^NO decay observed before the addition of the *bcc-aa*_3_ is due to the non-enzymatic reaction of ^•^NO with the reductants. Additionally, the initial fast drop in the ^•^NO concentration detected immediately after the addition of the enzyme is probably due to ^•^NO binding to the *bcc-aa*_3_. The ^•^NO-consuming activity of the *bcc-aa*_3_ under anaerobic conditions at ~8 µM ^•^NO added appeared to be about 3 mol ^•^NO × (mol *bcc-aa*_3_)^−1^ × min^−1^ [57] (Table 3). As one can see, this is ~100 times lower than that observed under aerobic turnover conditions. A similar activity was also reported previously for such heme–copper oxidases as the *ba*_3_ and *caa*_3_ from *Thermus thermophilus* [145], the *bo*_3_ from *E. coli* [146], the *cbb*_3_ from *Pseudomonas stutzeri* [147] and *R. sphaeroides* [148]. Notably, the mitochondrial beef heart *aa*_3_-type oxidase does not catalyze the anaerobic degradation of ^•^NO [149].

For the *ba*_3_ oxidase from *T. thermophilus* it was directly shown by gas chromatography that the end product of the catalytic ^•^NO decay under reducing anaerobic conditions is nitrous oxide (N_2_O), i.e., the ^•^NO reductase activity takes place [145]. It is reasonable to suggest that this is also the case for the other bacterial oxidases, which were reported to degrade ^•^NO under the same conditions [57,146,148]. The reaction mechanism could resemble that used by native bacterial ^•^NO reductases. Both mechanisms, however, are still under debate [150,151]. In general, two ^•^NO molecules react with the fully reduced BNC of the oxidase yielding one molecule of N_2_O as the end product, with the formation of the hyponitrite species as a transient intermediate. For more details, see Figure 23 in [151].

Since the ^•^NO reductase activity measured in some bacterial oxidases is not too high and the conditions requested hardly often occurs in vivo, we do not expect that this contributes significantly to microbial defense mechanisms against ^•^NO-induced stress.

### 3.2. bd-Type Oxidases Confer Bacterial Resistance to ^•^NO

Evidence is accumulating that in at least some pathogenic bacteria, cytochrome *bd* is involved in their defense against ^•^NO-induced stress. Jones-Carson et al. examined the role of the two major terminal oxidases of *Salmonella* Typhimurium, the heme–copper cytochrome *bo*_3_ (encoded by the *cyoABCD* operon) and cytochrome *bd* (encoded by the *cydAB* operon), in its antinitrosative defensive system [152]. The authors compared growth rates of the wild-type strain, Δ*cyoABCD,* and Δ*cydAB* mutants in LB broth supplemented with 5 mM DETA NONOate. The latter is the ^•^NO donor that at the added concentration produced a stable flux of 5 μM ^•^NO during the experiment. In contrast to the wild-type and Δ*cyoABCD* strains, the Δ*cydAB* mutant appeared to be hypersusceptible to ^•^NO as manifested by the extended lag phase following the DETA NONOate addition. Jones-Carson et al. also compared the rates of respiration in the wild-type, Δ*cyoABCD,* and Δ*cydAB* bacterial cultures treated with 50 μM spermine NONOate. The O_2_ consumption activity of the Δ*cydAB* mutant was much more sensitive to spermine NONOate as compared to that of the wild-type bacteria. Additionally, unlike the wild-type and Δ*cyoABCD* cells, the O_2_ consuming activity of the Δ*cydAB* cells did not improve over time following the addition of spermine NONOate. Cytochrome *bd* was reported to add to the ^•^NO-detoxifying activity of the flavohemoglobin Hmp that converts ^•^NO into NO_3_^−^. Both Hmp and the *bd* oxidase contribute to similar extents to *S.* Typhimurium pathogenesis. Furthermore, there is a substantial degree of independence between these two proteins in *S.* Typhimurium pathogenesis. It is suggested that low O_2_ levels in mice favor ^•^NO detoxification by cytochrome *bd* whereas high O_2_ tension favor Hmp as the ^•^NO-detoxifier. Bacteria may experience different O_2_ and ^•^NO levels as the inflammatory response evolves over time during the infection. Therefore, *S.* Typhimurium may preferentially use Hmp or the *bd* oxidase according to the availability of O_2_ and ^•^NO. Thus, cytochrome *bd*, along with Hmp, is an important component of the antinitrosative defensive system of *S.* Typhimurium [152].

Shepherd et al. examined the relative contribution of cytochrome *bd*-I (CydAB), Hmp, the flavorubredoxin NorVW, the nitrite reductase NrfA, and the iron–sulfur cluster repair protein YtfE to the ^•^NO-tolerance mechanisms in a multidrug-resistant uropathogenic *E. coli* (UPEC), strain EC958 [153]. For this purpose, the authors mutated the *cydAB*, *hmp*, *norVW*, *nrfA* and *ytfE* genes in EC958. Growth rates of wild-type EC958, and *cydAB*, *hmp*, *norVW*, *nrfA* and *ytfE* mutants were measured following the addition of the ^•^NO-releaser NOC-12 under microaerobic conditions. It turned out that mutation of *cydAB* and *hmp* confers the highest sensitivity to ^•^NO. Furthermore, the Δ*cydAB* mutant displayed increased sensitivity to neutrophil killing, reduced survival within primed macrophages, and an attenuated colonization phenotype in the mouse bladder. The fact that deletion of *cydAB* impairs survival in a mouse model suggests that the *bd* oxidase-dependent respiration under nitrosative stress conditions is a key factor for host colonization. Thus, the UPEC cytochrome *bd*-I provides the greatest contribution to ^•^NO tolerance and host colonization at low O_2_ tensions and is of major importance for the accumulation of high microbial loads in the course of infection of the urinary tract [153].

Beebout et al. reported that cytochrome *bd* of UPEC (*E. coli* cystitis isolate UTI89) is highly expressed in biofilms and that loss of the *bd*-oxidase-expressing subpopulation impairs barrier function and reduces the abundance of extracellular matrix [154]. The authors hypothesized that cytochrome *bd* is preferentially expressed in the UPEC biofilm because the enzyme provides protection against nitrosative stress. The addition of the ^•^NO donor NOC-12 to planktonic cultures was found to significantly reduce the growth rate of the *ΔcydAB* mutant: the doubling time increased from 37 to 106 min after the treatment. This finding suggests that during aerobic growth the *bd* oxidase serves as an ^•^NO sink that reversibly sequesters ^•^NO. This protects respiration mediated by cytochrome *bo*_3_ which is a proton pump that is more efficient at transducing energy but susceptible to irreversible inhibition by ^•^NO. Beebout et al. proposed that cytochrome *bd*-expressing subpopulations in UPEC are critical for withstanding such harmful metabolic by-products as ^•^NO while in the biofilm state [154].

Consistently, ^•^NO caused more significant growth inhibition in non-pathogenic *E. coli* strains lacking cytochrome *bd* as compared to cytochrome *bo*_3_-deficient ones [155]. In *Shewanella oneidensis*, the *bd* oxidase provides tolerance to nitrite rather than ^•^NO, but this is an exceptional case [156]. A protective role of cytochrome *bd* against ^•^NO stress also agrees with the expression of this enzyme in *E. coli* [154,157,158], *S.* Typhimurium [152], *Staphylococcus aureus* [159], *Bacillus subtilis* [160], and *M. tuberculosis* [161] in response to ^•^NO. Interestingly, in *M. tuberculosis*, the *bd* oxidase was reported to be necessary for optimal respiration at acidic pH as the *bcc-aa*_3_ supercomplex is markedly inhibited under these conditions [162].

**Figure 6:**
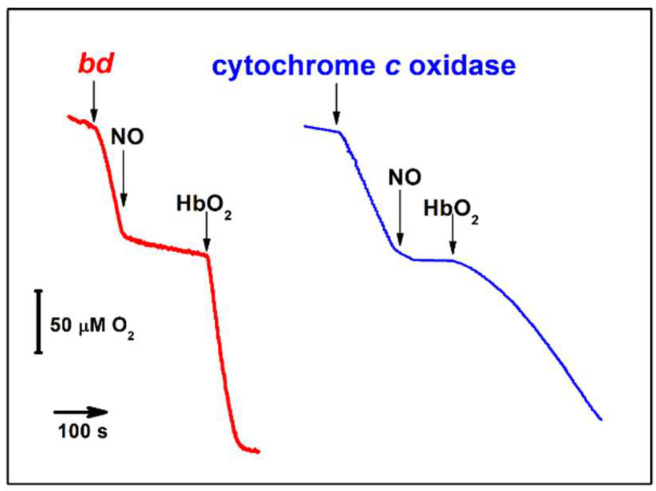
Activity recovery from ^•^NO inhibition of *E. coli* cytochrome *bd*-I and beef heart cytochrome *c* oxidase. Shown are time courses of O_2_ consumption by the enzymes. ^•^NO inhibits the enzymatic O_2_ consumption. Oxyhemoglobin (HbO_2_) scavenges rapidly all free ^•^NO that leads to reversal of ^•^NO inhibition. Modified from [111] with permission.

**Figure 7:**
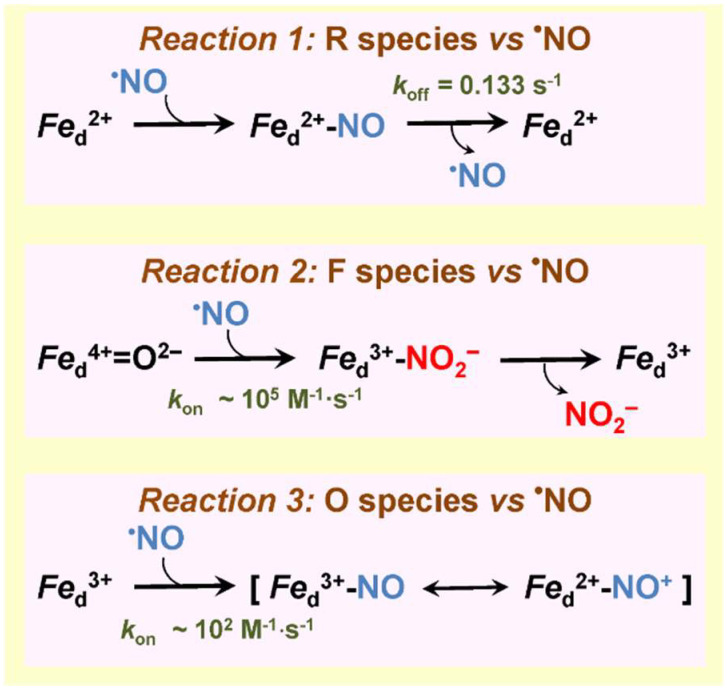
Reactions of ^•^NO with different cytochrome *bd* species.

Like most heme–copper oxidases tested (see Section 3.1), the *bd*-type oxidases from non-pathogenic *E. coli* and *A. vinelandii* are rapidly inhibited by ^•^NO [142]. This was demonstrated on the level of both the purified enzymes from these bacteria [142] and the *E. coli* cells lacking cytochrome *bo*_3_ [155,163]. The inhibition is reversible with the *IC*_50_ value of 100 nM ^•^NO for the purified *bd* oxidases from *E. coli* and *A. vinelandii* at 70 μM O_2_ in the assay medium [142] (Table 3). Unlike some heme–copper oxidases (see Section 3.1.2), cytochrome *bd* does not exhibit a measurable ^•^NO reductase activity under anaerobic conditions. The question arises as to if cytochrome *bd* is quickly inhibited by submicromolar concentrations of ^•^NO and unable even scavenge this RNS via ^•^NO reductase-like reaction, how can it serve as one of the key mechanisms for protecting bacteria against nitrosative stress? Phenomenologically, the answer to this question can be obtained by comparing the kinetic profiles of activity recovery from ^•^NO inhibition following the addition of the ^•^NO scavenger oxyhemoglobin (HbO_2_) for the *bd* oxidase and the mitochondrial cytochrome *c* oxidase (Figure 6). Upon ^•^NO depletion in solution by HbO_2_, the recovery is significantly faster in cytochrome *bd* than in the mitochondrial oxidase under similar experimental conditions [142,164]. However, what molecular mechanisms underlie such a rapid recovery of activity in the case of the *bd* oxidase? Studies of the interaction of ^•^NO with different cytochrome *bd* species made it possible to shed light on the molecular mechanisms [142,143,165,166]. ^•^NO binds at the level of the heme *d* active site. The reaction occurs if heme *d* is in the ferrous, ferryl, or ferric state. The rate of ^•^NO binding to the ferrous uncomplexed heme *d* (R species) has never been measured. One may expect that its value (*k*_on_) is comparable with those for the binding of CO and O_2_ to the fully reduced enzyme, i.e., in the range of 10^8^ to 10^9^ M^−1^·s^−1^ [101]. The reaction yields the nitrosyl ferrous heme *d* adduct (Figure 7, reaction 1) [72]. It turned out that the rate of ^•^NO dissociation from heme *d*^2+^ (*k*_off_) in the purified fully reduced cytochrome *bd*-I of *E. coli* is unusually high, 0.133 s^−1^ [143] (Table 3). A similar value (0.163 s^−1^) was later reported for membrane preparations of *E. coli* mutant strain RKP4544 devoid of cytochrome *bo*_3_ [155]. This *k*_off_ value is about 30 times higher than that for ^•^NO dissociation from ferrous heme *a*_3_ in the mitochondrial cytochrome *c* oxidase [164]. Furthermore, the ^•^NO off-rate for cytochrome *bd* is faster than that detected for almost all heme proteins. Such a high ^•^NO dissociation rate obviously explains why after ^•^NO-inhibition the activity of cytochrome *bd* is restored much faster than that of the mitochondrial oxidase (Figure 6). The reaction of ^•^NO with the *A. vinelandii* cytochrome *bd* in the ferryl state (F species) is fast (~10^5^ M^−1^·s^−1^) and likely produces the oxidized enzyme with nitrite bound at ferric heme *d* (Figure 7, reaction 2) [165]. This is about 10 times faster than the same reaction for the mitochondrial cytochrome *c* oxidase (~10^4^ M^−1^·s^−1^) [138,167]. Then, NO_2_^–^ likely escapes from heme *d*^3+^ to the bulk phase, but the off rate for nitrite has to be determined. Since intermediate F is highly populated in turnover [105], we think that the rapid oxidation of ^•^NO into NO_2_^–^ by cytochrome *bd* also contributes to the mechanisms of bacterial resistance to ^•^NO. The reaction of ^•^NO with ferric heme *d* in the purified fully oxidized cytochrome *bd*-I of *E. coli* (O species) proceeds with *k*_on_ of ~ 10^2^ M^−1^·s^−1^ yielding a nitrosyl adduct, *d*^3+^–NO or *d*^2+^–NO^+^ (Figure 7, reaction 3) [166]. The reaction is rather slow and the O species is not a catalytic intermediate of cytochrome *bd* [168] therefore it barely contributes to mechanisms of ^•^NO-inhibition or ^•^NO tolerance. Thus, we can conclude that the *bd* oxidase confers ^•^NO resistance to bacteria due to (i) extraordinary high ^•^NO off-rate and (ii) the ability to rapidly convert ^•^NO into NO_2_^–^ in turnover.

## 4. Peroxynitrite and Bacterial Terminal Oxidases

The study of the interaction of peroxynitrite with bacterial terminal oxidases is at the very initial stage. To date, the only bacterial oxidase that has been studied for the reaction with this highly reactive toxic compound is cytochrome *bd*-I from *E. coli* [109,169]. Earlier, the interaction of the eukaryotic heme–copper oxidase, the *aa*_3_-type cytochrome *c* oxidase isolated from bovine heart mitochondria, with ONOO^−^ was investigated [170]. It was shown that the mitochondrial enzyme when solubilized or in proteoliposomes is irreversibly damaged by ONOO^−^ (Table 4). At concentrations of less than 20 µM ONOO^−^ significantly raises the enzyme’s *K*_m_ for O_2_. This effect was tentatively explained by the nitration of some tyrosine residues [137]. At higher concentrations ONOO^−^ was reported to decrease the *V*_max_. The ONOO^−^-induced lowering of the *V*_max_ could be due to both the destruction of the Cu_A_ site in cytochrome *c* oxidase, and the irreversible loss of the 830-nm absorption band characteristic of the oxidized Cu_A_ was observed [170], and the degradation of hemes *a* and *a*_3_.

**Figure 8:**
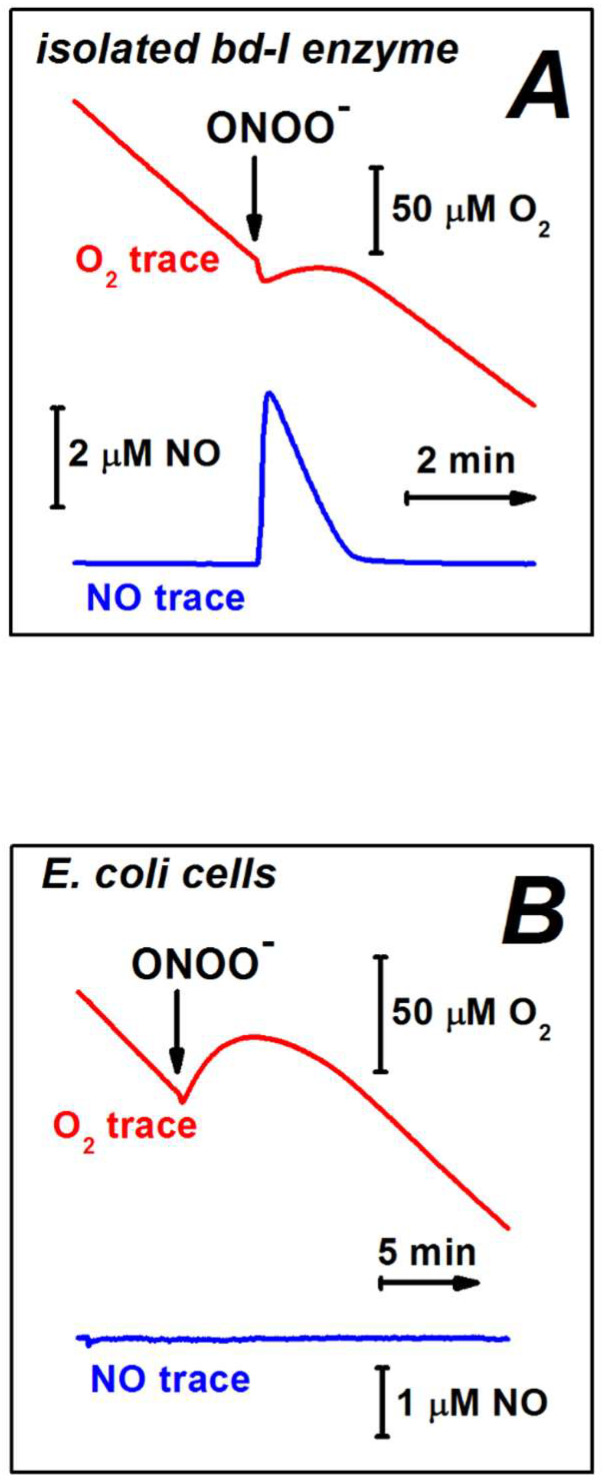
The effect of peroxynitrite on the O_2_ consumption by cytochrome *bd*-I from *E. coli*. (**A**) 50 µM ONOO^−^ was added to the isolated enzyme in the presence of 10 mM ascorbate and 0.5 mM TMPD. (**B**) 80 µM ONOO^−^ was added to the cell suspension of the *E. coli* strain GO105/pTK1 overexpressing cytochrome *bd*-I. The ^•^NO concentration was measured in parallel. Modified from [169] with permission.

Borisov et al. studied amperometrically the effect of ONOO^−^ on the O_2_ consumption by the *E. coli* cytochrome *bd*-I at the level of the isolated detergent-solubilized enzyme and the *bd*-I overexpressing bacterial cells [169]. It turned out that in both cases, the O_2_ consumption by the *bd*-I oxidase is not inhibited by up to 0.1 mM ONOO^−^ (Figure 8, Table 4). The effect of higher ONOO^−^ concentrations was not tested. After the addition of ONOO^−^ a slight short-term generation of O_2_ was observed (Figure 8). This is likely due to the catalase-like activity of cytochrome *bd*-I that scavenges H_2_O_2_, a contaminant in the commercial ONOO^−^ or a product of the peroxynitrite degradation [109,113,114]. Furthermore, using the stopped-flow rapid mixing technique it was shown that the *bd*-I oxidase is able to catalyze scavenging of ONOO^−^. The kinetics of this reaction was measured [169]. In these experiments, the enzyme pre-reduced anaerobically with excess reducing agents, *N*,*N*,*N’*,*N’*-tetramethyl-*p*-phenylenediamine (TMPD), and ascorbate, was mixed with an air-equilibrated solution of ONOO^−^. The ONOO^−^ decomposition rate was determined at 310 nm. It was found that ONOO^−^ disappears with an observed rate constant that is proportional to the cytochrome *bd*-I concentration and increases with the TMPD concentration. Importantly, in control experiments, neither the protein nor the reductants tested independently reveal the decay of ONOO^−^ to a significant extent. The apparent turnover rate at which the *bd*-I oxidase, in turnover with O_2_ and excess TMPD and ascorbate, decomposes ONOO^−^, was estimated to be ~600 mol ONOO^−^ × (mol enzyme)^−1^ × min^−1^ [169] (Table 4). Since the rate constant was found to increase with the enzyme activity (the electron flux), in the bacterial cell in which cytochrome *bd*-I utilizes ubiquinol as the substrate, the peroxynitrite-decomposing activity may be even higher. For instance, a turnover number of cytochrome *bd*-I is about seven times higher when the reducing system is ubiquinone-1 plus DTT as compared to that for TMPD plus ascorbate [168]. If the peroxynitrite-neutralizing activity of the *bd*-I oxidase is proportional to the electron flux, its apparent turnover rate in the *E. coli* cell could be as high as ~4200 mol ONOO^−^ × (mol enzyme)^−1^ × min^−1^. To summarize, the *E. coli* cytochrome *bd*-I in the catalytic steady state is not only resistant not ONOO^−^, but also capable of decomposing this highly reactive cytotoxic effector, thus serving as an important detoxifier of ONOO^−^ in vivo.

**Figure 9:**
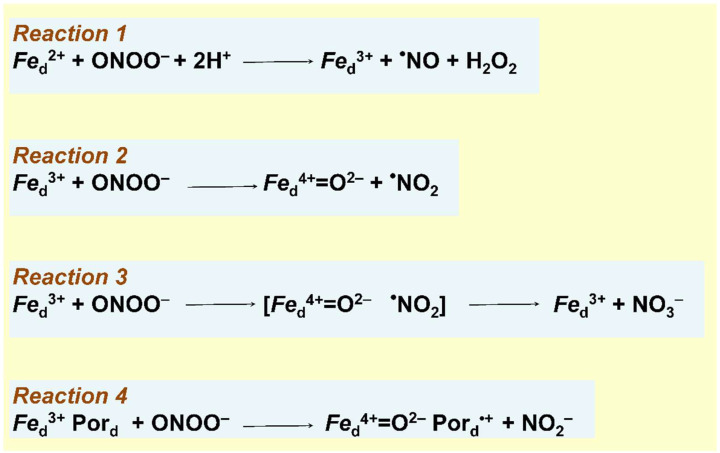
Possible mechanisms of the peroxynitrite decomposition catalyzed by cytochrome *bd*-I from *E. coli*.

A possible mechanism of the peroxynitrite decomposition catalyzed by the *bd*-I enzyme has never been proposed. We assume that the most likely site for the reaction is the high-spin heme *d*. We may suggest at least four possible reaction mechanisms. The fact that the addition of ONOO^−^ to the isolated *bd*-I protein in turnover with O_2_ and reductants resulted in the production of ^•^NO [169] (Table 4) points out that ^•^NO could be the main product. If this is the case, a one-electron reduction of ONOO^−^ to ^•^NO and H_2_O_2_ by the ferrous heme *d* may occur (Figure 9, reaction 1). If so, at least part of the H_2_O_2_ transiently generated following the addition of ONOO^−^ to the enzyme is also the main reaction product. There are two observations that are not consistent with the mechanism proposed. According to the reaction scheme (Figure 9, reaction 1), the decay of one molecule of ONOO^−^ added should generate one molecule of ^•^NO. In the experiments, however, the amount of ^•^NO produced was approximately 12 times less than the amount of ONOO^−^ added. In addition, no ^•^NO production was detected with the ONOO^−^–treated cells while the short-term generation of H_2_O_2_ is in place (Figure 8). The latter two findings indicate that the ^•^NO produced in the case of the isolated enzyme might be a secondary product, possibly non-enzymatic because the formation of ^•^NO was also observed in the absence of the protein, albeit to a lesser extent [169].

It was reported that ONOO^−^ generates Compound II (*Fe*^4+^ = O^2−^) in myeloperoxidase, lactoperoxidase, and catalase, and Compound I (*Fe*^4+^ = O^2−^ Por^•+^, where Por^•+^ is a porphyrin radical) in horseradish peroxidase [171,172]. Since these are ferriheme (*Fe*^3+^) enzymes, in these reactions ONOO^−^ serves as a one-electron and two-electron oxidant, respectively. We, therefore, suggest that in cytochrome *bd*-I ONOO^−^ also could react with the ferric heme *d*, (e.g., to the O^1^ catalytic intermediate, see Figure 2). In the case of one-electron oxidation heme *d*^3+^ is converted to Compound F (analog of Compound II, see Figure 2) with the concomitant release of ^•^NO_2_ from ONOO^−^ (Figure 9, reaction 2).

It is also possible that the ferric heme *d* catalyzes the isomerization of peroxynitrite to nitrate (NO_3_^–^). If so, Compound F and ^•^NO_2_ are transient reaction intermediates, not the final products (Figure 9, reaction 3). The fact that certain iron (III) porphyrins are capable of catalyzing the isomerization of ONOO^−^ to NO_3_^–^ [173] is in agreement with this hypothesis.

In the case of two-electron oxidation heme *d*^3+^ is converted to Compound F* (analog of Compound I, see Figure 2) with the co-production of NO_2_^−^ from ONOO^−^ (Figure 9, reaction 4). It is worth noting that microbial and mammalian peroxiredoxins catalyze detoxification of peroxynitrite via its two-electron reduction to nitrite [174,175].

## 5. Concluding Remarks

Usually, terminal oxygen reductases of bacterial respiratory chains are strongly inhibited by nitric oxide and peroxynitrite. However, some of the respiratory enzymes, such as the mycobacterial *bcc*-*aa*_3_ supercomplex and *bd*-type oxidases, confer resistance to RNS, thereby contributing to microbial pathogenicity. An understanding of the molecular mechanisms of bacterial pathogenicity is essential for the development of new strategies to combat infectious diseases. In this regard, it would be interesting to figure out the reaction mechanisms underlying *bcc*-*aa*_3_ supercomplex-mediated ^•^NO detoxification and importantly, whether this unique property of the mycobacterial enzyme is shared by other *aa*_3_-type oxidases, eventually complexed with the *bc*_1._ The interest in *bd*-type oxidases is increasing due to their peculiar enzymatic abilities, stress tolerance, and importance to pathogens—features that merit more in-depth functional and structural studies. Determination of cytochrome *bd* structure from different microorganisms would help in the characterization and rational design of selective inhibitors of these oxidases. Based on already published 3D structures of *bd*-type oxidases, one of the main challenges in the structure-driven design of quinone substrate-like inhibitors is expected to be the high flexibility of the N-terminal part of the quinol binding site called the Q-loop. Another promising direction for future research is the study of the effect of RNS on the anaerobic terminal reductases and other bioenergetic enzymes in anaerobic pathogenic bacteria. All in all, the development of next-generation antibiotics selectively targeting the RNS-insensitive respiratory complexes in pathogens may reduce their impact on human health and social development.

## Figures and Tables

**Table 1:** Major enzyme complexes of the mammalian mitochondrial electron transport chain.

Enzyme Complex	Electron Donor	Electron Acceptor	Energy Currency Produced
Complex I	NADH	ubiquinone	proton motive force (PMF)
Complex II	succinate	ubiquinone	none
Complex III	ubiquinol	ferricytochrome *c*	PMF
Complex IV	ferrocytochrome *c*	O_2_	PMF

**Table 2:** Major enzyme complexes found in aerobic bacterial electron transport chains.

Enzyme Complex	Electron Donor	Electron Acceptor	Energy Currency Produced
NDH-1	NADH	quinone	PMF
NDH-2	NADH	quinone	none
NQR	NADH	quinone	Na^+^ motive force
Complex II	succinate	quinone	none
Complex III	quinol	ferricytochrome *c*	PMF
Heme–copper oxidases (*aa*_3_, *caa*_3_, *bo*_3_, *cbb*_3_, *ba*_3_)	ferrocytochrome *c* or quinol	O_2_	PMF
Cytochrome *bcc*-*aa*_3_ supercomplex	quinol	O_2_	PMF
Cytochrome *bd* (*bd*-I, *bd*-II)	quinol	O_2_	PMF
Cyanide insensitive *bd*-type oxidase (CIO)	quinol	O_2_	n.d.

**Table 3:** Overview of ^•^NO interactions with mycobacterial cytochrome *bcc-aa*_3_ supercomplex and *E. coli* cytochrome *bd*-I, respiratory enzyme complexes which contribute to mechanisms of bacterial resistance to ^•^NO.

Enzyme Complex	Inhibition by ^•^NO	^•^NO Degradation in Turnover	Anaerobic ^•^NO Degradation	^•^NO off-Rate	NO_2_^–^ off-Rate	Reference
Mycobacterial cytochrome *bcc-aa*_3_ supercomplex	No	Yes (~300 mol ^•^NO × (mol *bcc-aa*_3_)^−1^ × min^−1^)	Yes (~3 mol ^•^NO × (mol *bcc-aa*_3_)^−1^ × min^−1^)	n.d.	n.d.	[57]
*E. coli* cytochrome *bd*-I	Yes (*IC*_50_ = 100 nM ^•^NO at 70 μM O_2_)	No	No	0.133 s^−1^	n.d.	[142,143]

**Table 4:** Overview of ONOO^−^ interactions with bovine heart *aa*_3_-type cytochrome *c* oxidase and *E. coli* cytochrome *bd*-I.

Enzyme Complex	Inhibition by ONOO^−^	^•^NO Production after ONOO^−^ Addition	Short-Term Generation of O_2_ just after ONOO^−^ Addition	Direct Observation of ONOO^−^ Degradation in Turnover	Reference
Purified bovine heart *aa*_3_-type cytochrome *c* oxidase	Yes (irreversible damage to enzyme complex)	Yes	No	No	[170]
Purified *E. coli* cytochrome *bd*-I	No (up to 0.1 mM ONOO^−^)	Yes	Yes	Yes (~600 mol ONOO^−^ × (mol *bd*-I)^−1^ × min^−1^)	[169]

## Data Availability

Not applicable.
